# Concise Review on Clinical Applications of Conditioned Medium Derived from Human Umbilical Cord-Mesenchymal Stem Cells (UC-MSCs)

**Published:** 2018-07-01

**Authors:** Sushmitha Sriramulu, Antara Banerjee, Rosa Di Liddo, Ganesan Jothimani, Madhumala Gopinath, Ramachandran Murugesan, Francesco Marotta, Surajit Pathak

**Affiliations:** 1 Faculty of Allied Health Sciences, Chettinad Hospital & Research Institute(CHRI), Chettinad Academy of Research and Education(CARE), Kelambakkam, Chennai, 603 103, India; 2 Department of Pharmaceutical and Pharmacological Sciences, University of Padova, Padova, Italy; 3 ReGenera R&D International for Aging Intervention, Milano-Beijing, Italy-China, VCC Preventive Medical Promotion Foundation, Beijing, China

**Keywords:** Umbilical cord, Stem cells, Conditioned medium, Therapeutics, Isolation

## Abstract

In recent years, mesenchymal stem cells have provoked much attentiveness in the field of regenerative medicine because of their differentiation potential and the capability to facilitate tissue repair via the emancipation of biologically active molecules. They have gained interest because of their distinctive curative properties. Mesenchymal stem cells are isolated from the Wharton’s jelly part of umbilical cord possessing higher proliferation capacity, immunomodulatory activity, plasticity, as well as self-renewal capacity than the mesenchymal stem cells from various origins, and it is considered to be the best resource for allogeneic transplantation. The isolated umbilical cord-derived mesenchymal stem cells are cultured in the Dulbecco’s Modified Eagle’s Medium, and thereby it begins to release soluble factors into the medium during the period of culture which is termed as conditioned medium. This conditioned media has both differentiation capacity and therapeutic functions. Thus, it can be able to differentiate the cells into different lineages and the paracrine effect of these cells helps in replacement of the damaged cells. This medium may accord to optimization of diagnostic and prognostic systems as well as the generation of novel and targeted therapeutic perspectives.

## Introduction

 Mesenchymal stem cells (MSCs) are considered to be multipotent stromal cells that are derived from the mesoderm with self-renewal capacity ^1^. Friedenstein et al. first published his report on explaining the expansion of adherent and spindle shaped cells derived from human bone marrow ^2^. These MSCs have the ability to differentiate into various lineages which include osteocytes (bone cells), chondrocytes (cartilage cells), myocytes (muscle cells) and adipocytes (fat cells) ^3^. The usual functioning role of mesenchymal stromal cells is maintaining the tissue homeostasis by differentiating towards the tissue specific cell types, proliferation and also by emancipating the growth factors and immunoregulatory agents ^4^. Some recent evidence stipulates that the MSCs assist in tissue regeneration through hindrance or inhibition of unwanted immune reactions and impart growth factors rather than directly restoring the damaged cells ^5^.

These multipotent adult stem cells have been isolated from different tissues, including bone marrow, adipose tissue, umbilical cord, cord blood, amniotic fluid, peripheral blood, placenta, fetal lung, etc ^6^. These MSCs have immunosuppressive and anti-inflammatory effects, which might constitute an interesting source for curative applications. They also inhibit the maturation of dendritic cells, enhancement of anti-inflammatory functions and declination of the production of cytokines ^2^. Bone marrow is previously considered as the chief source of MSCs, but their number begins to decrease significantly with age and this has led to the discovery of numerous alternative sources. When compared to bone marrow-derived stem cells, the umbilical cord-mesenchymal stem cells (UC-MSCs) have less human leukocyte antigen-II (HLA-II) and major histocompatibility complex (MHC) class I molecules. These UC-MSCs have been proposed as a candidate for numerous clinical applications. The conditioned medium (CM) derived from MSCs was also found to have similar clinical applications as that of MSCs from the previous studies. So, it is imperative to understand the application and therapeutic properties of the CM.

**Human umbilical cord derived-mesenchymal stem cells (UC-MSCs)**

The MSCs are obtained from various umbilical cord (UC) compartments or from the complete UC. This UC generally possesses about four compartments as a source of MSCs: 

1. The whole UC is cut into minute pieces or fragments and thereby following the enzymatic digestion process or explant techniques. 

2. The Wharton’s jelly (WJ) MSCs is acquired by removal of the UC vessels (arteries and vein). The umbilical cord arteries (UCA) and veins (UCV) (umbilical vessels) can also be minced into minute fragments and then plated for the growth of MSCs. 

3. The sub-amnion part of the lining membrane is unfastened with a blade, and it is cut into minute pieces. 

4. The extracted vessels from the cord have to be tied at the two ends in a kind of loop. The loops will be presented in an enzymatic solution for a definite period of time to make the cells to dissociate from the perivascular part ^7^. 

Thus, these MSCs are generally isolated by enzyme digestion process as well as density gradient centrifugation. The isolated cells were plated in a complete culture medium with the supplements and maintained at 37°C under 5% CO_2_. Cell growth are analyzed by direct cell counts ^2^. 

**Figure 1:**
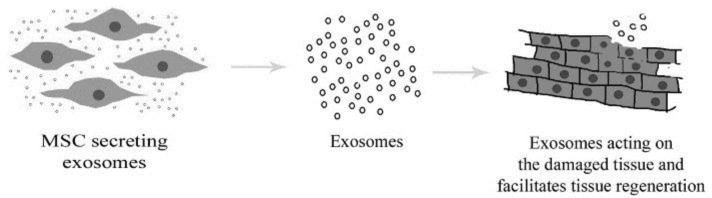
Paracrine effect of mesenchymal stem cells (MSCs) in tissue regeneration

Other than the general advantages such as painless collection approach and rapid self-renewal ability, UC-MSCs has displayed the capacity to differentiate into three different germ layers to accumulate in the damaged tissue or inflamed regions and to provoke tissue repair. These MSCs which are isolated from Wharton’s jelly of UC have higher proliferation capacity, immunomodulatory activity, plasticity as well as self-renewal ability than MSCs derived from various origins and are considered to be the best resource for allogeneic transplantation. These UC-MSCs are characterized by high safety, abundance, less immunogenicity and shorter amplification times. Most of the research studies show that these circulating MSCs are attracted to the sites of damage or inflamed regions where it undergoes tissue-specific differentiation (Figure 1).

**Conditioned medium from UC-MSCs**

**Figure 2:**
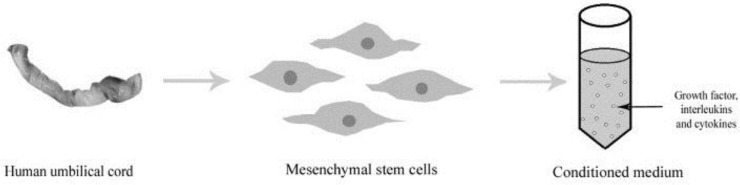
Collection of conditioned medium (CM) from human umbilical cord-mesenchymal stem cells (UC-MSCs)

The isolated UC-derived MSCs are cultured in the Dulbecco’s Modified Eagle’s Medium (DMEM) medium, and thereby it begins to emancipate certain soluble factors into the medium during the period of culture which is termed as CM (Figure 2).

These factors that are secreted thereby promote anti-oxidant, immunosuppressive and cell proliferative effects at the transplant regions. The CM has both differentiation capacity and therapeutic function. Thus, it can be able to differentiate the cells into different lineages and the paracrine effect of these cells helps in replacement of the damaged cells. This derived CM might intensify the positive effects of cell-based therapies. Since UC-CM contains increased levels of growth factors and chemokines, they normally accord to a chemoattractive environment of the adjacent tissues ^8^. The growth factors and cytokines secreted from adipose tissue-derived MSCs have been elucidated in previous studies (Table 1) ^9^.

**Table 1:** Growth factors, cytokines and chemokines secreted by human adipose tissue derived mesenchymal stem cells (MSCs) in the conditioned medium

**Factors**	**Role**
IL-6	It prevents differentiation, apoptosis induced by serum starvation and as well as enhances proliferation of undifferentiated MSCs.
VEGF	Plays a vital role in angiogenesis, post-natal homeostasis, and skeletal development and also regulates the differentiation of MSCs by modulating the levels of RUNX2 and PPARγ transcription factors.
Angiogenin	Interaction with endothelial cell induces broad range of cellular responses such as cellular proliferation, cell migration and tube formation, etc. and plays a vital role in stimulating angiogenesis.
MCP-1,3	These chemokines acts as a MSC homing factor, the overexpression of MCP signals the MSCs to identify the site of injured tissue and restores the damaged cells.
IGF-1	It plays a vital role in regulating neuronal functions. It suppresses neuronal apoptosis and reduces inflammatory reactions.
bFGF	Triggers FGF receptors which activate P13K/Akt signaling pathway. Thereby it induces human MSC migration via a FGFR/ P13K/Akt pathway.

## Discussion

**Clinical applications of conditioned medium**

**Figure 3:**
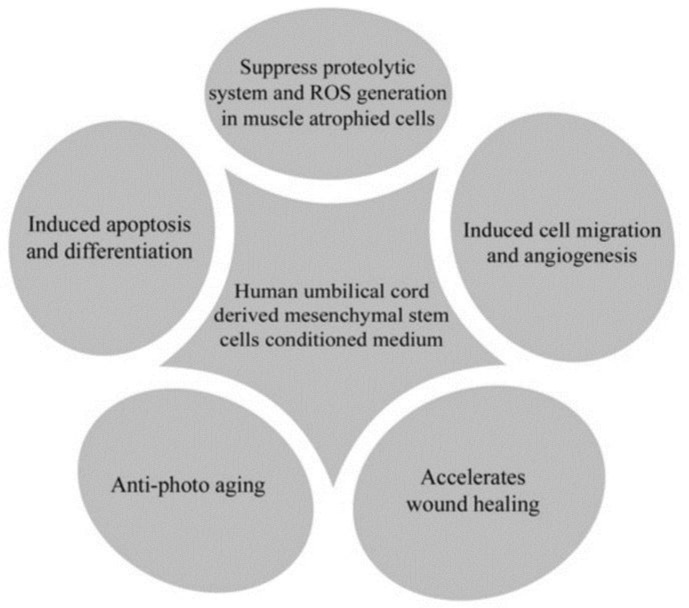
Clinical applications of conditioned medium (CM) derived from human umbilical cord-mesenchymal stem cells (UC-MSCs)

There are various therapeutic applications for CM, including anti-photoaging properties and accelerating the wound healing with fewer scars. It also helps to induce apoptosis and differentiation. Moreover, it plays an important role in inducing migration and angiogenesis, preventing muscle atrophy, possessing anti-fibrotic properties and regenerating capacities. It does help in suppressing proteolytic system and the ROS generation in muscle atrophied cells ^10^^-^^12^ (Figure 3). The diverse studies on the secreted factors derived from stem cells exhibited that the secreted soluble factors without the stem cells might provoke tissue repair in different conditions that involved in organ or tissue damage ^13^. Previous studies have examined the pro-angiogenic properties of UC-MSCs which have been described as exhibited *in vitro.* The impact of the CM on proliferation of human umbilical vein-derived endothelial EA.hy926 cells was quantified. The major alterations in the cell motility and undeviating migration were evaluated by the scratch-wound healing and trans-well chamber migration assays. Their studies indicated that VEGF-A independent paracrine activity mechanism and partially VEGF-A independent differentiation mechanism are generally implicated in the pro-angiogenic activity of UC-MSCs ^14^.

In a study, the regenerative impacts and the controlling mechanisms of UC-MSC derived CM were investigated in the atrophied muscles by utilizing an *in vivo* model, and their findings suggested that CM imparts a constructive stimulus to perpetuate the muscle status and function in atrophied muscles. Therefore, they concluded that the use of UC-CM will perform as a promising therapeutic approach for further expansion of cell-free therapies in muscle regeneration ^12^. MSCs derived from UC have also proven the cutaneous wound healing by means of paracrine mechanism. The dermal fibroblasts which were treated with the CM from UC-MSCs were found to additionally treat the wounds and displayed accelerated healing with fewer scars when compared to the controls ^10^. 

Another study examined the curative effect of UCMSC-CM on muscle-linked diseases by utilizing a dexamethasone (Dex)-induced muscle atrophy *in vitro* model. The expression of the muscle atrophy- linked proteins was found to be increased by around 50-70% when the L6 cells were exposed to Dex. The expression of muscle-specific proteins was in a way decreased by around 23–40%. In contrast, these L6 cells when co-treated with UCMSC-CM and Dex, the muscle atrophy-linked proteins shows a reduced expression in a UCMSC-CM dose-dependent manner and the expression of muscle-specific proteins was recovered to the near-usual levels. Besides, the ROS generation was found to be suppressed and expression of anti-oxidant enzymes was restored to a normal degree. These data implicates that the UCMSC-CM clearly has the capacity to treat muscle atrophy ^11^.

Previous studies have also demonstrated the anti-photoaging effects following chronic ultraviolet (UV) irradiation in both *in vitro* and *in vivo* of conditioned serum-free medium (SFM) derived from UC-MSCs were assessed. This UC-SFM had a restorative impact on the human dermal fibroblast proliferation and decreased UV-A induced cell death. Furthermore, UCMSC-SFM chunked UV-A obstruction of superoxide dismutase activity. The topical application of UCMSC-SFM to the mouse skin prior to UV irradiation chunked the hampering of superoxide dismutase and glutathione peroxidase activities and lessened the up-regulation of malonaldehyde. Thus, UCMSC-SFM protects against photoaging instigated by UV-A and UV-B radiation and is found to be a much assuring candidate for the skin anti-photoaging treatments^15^. Taken together, all the previous existing studies reveal that the use of UC-CM might be suitable for regenerative medicine.

## CONCLUSION

 MSCs are population of stem cells with greater self-renewal and multipotentiality. MSCs are potential seed cells in regenerative medicine and have been used for the treatment of various diseases. Stem-cell therapy, especially UC-MSCs, is a promising alternative to treat on-going tissue damage by resetting the underlying disease process through alteration of the mucosal immune response. Moreover, they are proven to settle in the inflamed sites to repair injured tissues. Recent findings suggest that MSC-CM have similar properties like MSCs and favourable antitumor characteristics as well. The understanding of these mechanisms and CM may contribute to the optimization of diagnostic and prognostic systems as well as the generation of novel and targeted therapeutic perspectives.
